# A Two-Step Real-Time PCR Method To Identify Mycobacterium tuberculosis Infections and Six Dominant Nontuberculous Mycobacterial Infections from Clinical Specimens

**DOI:** 10.1128/spectrum.01606-23

**Published:** 2023-06-28

**Authors:** Jungho Park, Naeun Kwak, Jong-Chan Chae, Eun-Jeong Yoon, Seok Hoon Jeong

**Affiliations:** a Division of Biotechnology, Chongbuk National University, Iksan, South Korea; b BioPark Diagnostics Inc., Seoul, South Korea; c Department of Biomedical Laboratory Science, College of Software and Digital Healthcare Convergence, Yonsei University MIRAE Campus, Wonju, South Korea; d Division of Antimicrobial Resistance Research, National Institute of Health, Korea Disease Control and Prevention Agency, Cheonju, South Korea; e Department of Laboratory Medicine, Yonsei University College of Medicine, Seoul, South Korea; f Research Institute of Bacterial Resistance, Yonsei University College of Medicine, Seoul, South Korea; University Paris-Saclay, AP-HP Hôpital Antoine Béclère, Service de Microbiologie, Institute for Integrative Biology of the Cell (I2BC), CEA, CNRS

**Keywords:** molecular diagnostics, *Mycobacterium tuberculosis*, nontuberculosis mycobacterium, real-time PCR

## Abstract

Tuberculosis (TB) is an ongoing threat to public health, and furthermore, the incidence of infections by nontuberculous mycobacteria (NTM), whose symptoms are not distinguishable from TB, is increasing globally, thus indicating a need for accurate diagnostics for patients with suspected mycobacterial infections. Such diagnostic strategies need to include two steps, (i) detecting the mycobacterial infections and, if the case is an NTM infection, (ii) identifying the causative NTM pathogen. To eliminate a false-positive TB diagnosis for a host vaccinated by the bacillus Calmette-Guérin (BCG) strain of Mycobacterium bovis, a new target specific for M. tuberculosis species was selected, together with the species-specific targets for the six dominant NTM species of clinical importance, i.e., M. intracellulare, M. avium, M. kansasii, M. massiliense, M. abscessus, and M. fortuitum. Using sets of primers and probes, a two-step real-time multiplex PCR method was designed. The diagnostic performance was assessed by using a total of 1,772 clinical specimens from patients with suspected TB or NTM infection. A total of 69.4% of M. tuberculosis and 28.8% of NTM infections were positive for the primary step of the real-time PCR corresponding to the culture within 10 weeks, and mycobacterial species of 75.5% of the NTM-positive cases were identified by the secondary step. The two-step method described herein presented promising results and similar diagnostic sensitivity and specificity to commercially available real-time PCR kits for detecting TB and NTM infections. The method also enabled the identification of mycobacterial species in three-quarters of NTM infection cases, thus providing a better treatment strategy.

**IMPORTANCE** Tuberculosis (TB) is an ongoing threat to public health. In addition, infection by nontuberculous mycobacteria (NTM) is a nonnegligible issue for global public health, with increasing incidences. Since the antimicrobial treatment strategy needs to be differed by the causative pathogen, a rapid and accurate diagnostic method is necessary. In this study, we developed a two-step molecular diagnostic method using clinical specimens of TB and NTM infection-suspected patients. The diagnostic power of the new method using the novel target was similar to the widely used TB detection kit, and, among the NTM-positive specimens, three-quarters of the NTM species were able to be identified. This simple and powerful method will be useful as it is, and it could be applied easily to a point-of-care diagnostic apparatus for better application to patients, especially those living in developing countries.

## INTRODUCTION

Tuberculosis (TB), an infectious disease caused by the Mycobacterium tuberculosis complex (MTC), was the 13th leading cause of death worldwide in 2021 and the second leading cause of death induced by communicable disease after COVID-19 (1, 2). Globally, many efforts are being made to provide proper treatments for TB patients and to prevent infection and transmission, including the development of rapid diagnostic methods and novel drugs for therapy. Such endeavors saved 66 million lives between 2000 and 2020, and the incidence of TB decreased by approximately 2% per year between 2015 and 2020 (2). Due to the continuing threats of TB to public health, efforts to control mycobacterial diseases have mainly focused on TB. However, infections by nontuberculous mycobacteria (NTM) are not negligible.

A significant proportion of patients are infected by NTM, and a recent report of global epidemiology for NTM infection indicated its growing incidence (3). NTM infection can be caused by various mycobacterial species, and the therapeutic strategy varies based on the causative pathogen. Due to the nonspecific pulmonary symptoms of the disease, failure to recognize NTM infections is common (4). Misidentification of the species is critical for misconception of the therapeutic strategy, and inappropriate empirical treatment leads to lengthy and expensive treatment and may result in treatment failure with the development of antimicrobial resistance (5). For instance, clarithromycin and other macrolides are never useful for patients infected by Mycobacterium abscessus, which is intrinsically resistant to macrolide antimicrobials. People at risk for NTM infection include elderly individuals with a medical history of lung disease and immunocompromised patients; NTM infections are common in developed countries (6).

In the algorithm for testing specimens from patients with suspected NTM infection, two major steps are necessarily included to set a proper therapeutic strategy, (i) detecting TB and/or NTM infections and, if the case is an NTM infection, (ii) species identification of the causative NTM pathogen. The tuberculin skin test, using *in vivo* sensitization by the pathogen, is one of the most widely used methods to determine the present and past historical TB of patients. However, the method often results in false-positive outcomes for the host vaccinated by the bacillus Calmette-Guérin (BCG) strain of M. bovis (7). Pure culture, together with acid-fast bacillus (AFB) smear microscopy, is considered the gold standard for diagnosis; however, the long turnaround time, low sensitivity, and difficulties in quality control of the test limit its sole use in modern clinical laboratories (8). To date, the molecular diagnostic method, i.e., PCR, is the most preferred technique for TB diagnosis, and the internal transcribed spacer region of the IS*6110* element specific for the M. tuberculosis complex is the most common target for detection (9). While TB can be caused by any member of the M. tuberculosis complex, the pathogenic ability of each species belonging to the M. tuberculosis complex appears to display host restriction. For instance, M. bovis, the cause of TB in cattle, rarely causes TB in human beings due to the different immune responses among hosts (10). The well-known M. bovis BCG strain, which had been attenuated by serial culture, was used as a live attenuated vaccine. Since the M. bovis BCG strain has one or two copies of the IS*6110* element (11), vaccination could lead to a false-positive result for PCR experiments, even though this is not the infection case.

In this study, we developed a nucleic acid amplification-based two-step molecular diagnostic method for TB and NTM infections, which primarily detects TB devoid of false-positive results by BCG vaccination and then uses clinical specimens to identify the top six prevalent NTM species in 2 h. Since the method is simple and rapid, it will be useful as a molecular diagnostic method, and furthermore, it could be applied to a point-of-care diagnostic apparatus for better application to patients, especially those living in developing countries.

## RESULTS

### Designing the primers and probes targeting M. tuberculosis and NTM.

For the NTM ID step, the top six dominant NTM species, M. abscessus, M. avium, M. intracellulare, M. kansasii, M. massiliense, and M. fortuitum, were determined based on a recent epidemiology study conducted in Seoul, South Korea (12). By using the reference genomes of target mycobacterial species, including M. tuberculosis and M. bovis, and the GenBank standard database of nucleotide collection mycobacterial species, the coding DNA sequence (CDS) for colonic acid biosynthesis glycosyltransferase conserved in M. tuberculosis as a core gene, but not in M. bovis or in NTM species, was selected for the target specific for M. tuberculosis. For the universal NTM species, the *rpoB* gene for the RNA polymerase beta subunit, conserved among NTM species, including others than the six dominant species, but not in the M. tuberculosis complex, was chosen. For the targets specific to the six dominant NTM species for the NTM identification step, the *erm*(41) gene for erythromycin ribosome methyltransferase in M. abscessus, the *hsp70* gene for heat shock protein in M. avium, the *rpoB* gene for RNA polymerase beta subunit in both M. massiliense and M. fortuitum, the *coi* gene for cytochrome *c* oxidase subunit I in M. intracellulare, and the internal transcribed spacer (ITS) region in M. kansasii were used to design the specific primers and probes. Conserved and varied regions were analyzed by multiple alignment, and possible false-positive and false-negative reactions were estimated by using BLAST against both databases of nucleotide collection and RefSeq genome for mycobacterial species by the criteria of 90% coverage and 80% identity. Evaluation of each set of primers and probes through actual reaction was carried out by using the total DNA of MTC and NTM species. An internal control for the reaction was designed artificially by considering the high G+C contents of the common genome of mycobacterial species, and the primers and probe were designed following the characteristics of other primers and probes to fit any of the three multiplex PCRs. The sets of primers and fluorescence-labeled probes for three multiplex PCRs were finalized as presented in Table 1.

**TABLE 1:** Sequences, concentrations, and fluorophores of the oligonucleotides contained in the multiplex qPCR assay

Purpose and target^*a*^	Oligonucleotide sequence (5′–3′)
M. tuberculosis and NTM detection	
M. tuberculosis	
Forward primer	CATCGCGCACGCATCGGG
Reverse primer	TCCACGGCCTGGGCTACCA
Probe (FAM-TAMRA)^*b*^	CACTCCGCCGATCGTTTTTCC
NTM	
Forward primer	CGGCTTCTCCGAGATCATGATG
Reverse primer	TCCAGYAGGGYCTGSGCS
Probe (HEX-TAMRA)	TGCTSGACATCTACCGVAARC
NTM identification	
M. abscessus	
Forward primer	GGTTTGCCGAGGAAGATGTCC
Reverse primer	ATCAGTGCGCTGGTGACTTGG
Probe (HEX-TAMRA)	GACCTGCTCGCCTTCCGG
M. avium	
Forward primer	CATCACCGCCAAACGGGAGT
Reverse primer	CTGACCACCACCTCGTTGGA
Probe (TEXASRED-BHQ2)	GTGCTCGTCTACGACCTCG
M. kansasii	
Forward primer	ATGGATGCGTTGCCCTACGG
Reverse primer	GCCCTTAGACACTTACAAACACAAA
Probe (CY5-BHQ2)	GTGTTCTTTTGTGCAATTTTATTCT
M. massiliense	
Forward primer	CCCGAGTGGGCGCAGAAC
Reverse primer	CGCGGGCACCGTCGAACA
Probe (HEX-TAMRA)	AGGACCTGCAGTCGGCTCC
M. intracellulare	
Forward primer	CAACAGTCATCGTCACAGCCG
Reverse primer	CCAGAATGCCCATCACCAGGA
Probe (ROX-TAMRA)	AAAGGCTGGGTCCAGGGTGT
M. fortuitum	
Forward primer	GGCTCGTCGGTGCGGATGA
Reverse primer	CTCGATCGGGGAGAGCTCTT
Probe (CY5-BHQ2)	GATCGCGGCGAGACCGAC
Internal control	
An artificial sequence^*c*^ for internal control	
Forward primer	GAGTAGGGAGTCAAGGCGAG
Reverse primer	GTTGGGACTCTCCCAAGTGG
Probe (FAM-TAMRA)	CTCCCCATGCACACCCCAG

a Target genes for each mycobacterial species are the gene for glycosyltransferase in M. tuberculosis, the *rpoB* gene in NTM, the *erm*(41) gene in M. abscessus, the *hsp70* gene in M. avium, the internal transcribed spacer region in M. kansasii, the *rpoB* gene in M. massiliense, the gene for nitric oxide reductase large subunit in M. intracellulare, and the *rpoB* gene in M. fortuitum.

b The pair of fluorophore (5′) and quencher (3′) of each probe is indicated in parentheses.

c Internal control template sequence (5′ to 3′), GGGGAGTAGGGAGTCAAGGCGAGGACCCCTGAGAGGGGTCTCCCCATGCACACCCCAGAGGCGTGCCACTTGGGAGAGTCCCAACGCC.

### Analytical performance.

Optimal fluorescence thresholds were taken through the common practice that they should be positioned on the lower half of the fluorescence accumulation curve plot from the 10-fold diluents, crossing most, if not all, fluorescence being detected on the exponential curve on a logarithmic scale. The baseline was set to the automatic function of the instrument, and the fluorescence threshold value was chosen to determine the cycle threshold value (*C_T_*) for each target to take the positive reaction, not the negative ones.

**FIG 1:**
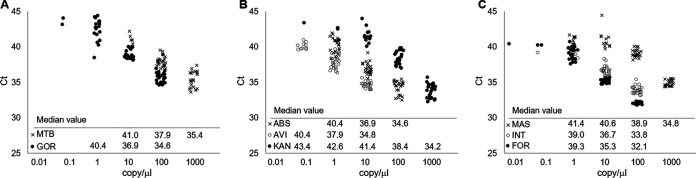
Distribution of the *C_T_* values at the total DNA copy per microliter with the median *C_T_* values for each reaction for the primary MTB/NTM detection step (A) and the NTM identification step (B and C). MTB, M. tuberculosis; GOR, M. gordonae; ABS, M. abscessus; AVI, M. avium, KAN, M. kansasii; MAS, *M. massiliense*; INT, M. intracellulare; FOR, M. fortuitum.

The analytical 95% limit of detection (LOD) was determined from four independent tests using total DNA of reference mycobacterial strains, six replicates of each 10-fold diluent, from 1 × 10^−2^ copies/μL to 1 × 10^3^ copies/μL (Fig. 1). Total DNA from M. tuberculosis, M. bovis, and M. gordonae, a representative NTM species, was used for the primary step for M. tuberculosis and NTM detection, and DNA from M. abscessus, M. avium, M. kansasii, M. massiliense, M. intracellulare, and M. fortuitum was used for both reactions for the secondary step for NTM identification.

Median *C_T_* values at each diluent of M. tuberculosis DNA were 35.4 at 1,000 copies/μL, 37.9 at 100 copies/μL, and 41.0 at 10 copies/μL, and those of M. gordonae were 35.5 at 100 copies/μL, 38.6 at 10 copies/μL, 42.8 at 1 copies/μL, and 43.6 at 0.1 copies/μL (Fig. 1). Finally, the calculated LOD values of M. tuberculosis and NTM, which was represented by M. gordonae, were 169.8 copies/μL and 1.6 copies/μL, respectively. Notably, M. bovis was never detected until 1,000 copies/μL of the template. The LOD values were 0.1 to 160.7 copies/μL for the six NTM species as 29.1 copies/μL for M. abscessus, 1.2 copies/μL for M. avium, 160.7 copies/μL for M. kansasii, 98.9 copies/μL for M. massiliense, 3.0 copies/μL for M. intracellulare, and 0.1 copies/μL for M. fortuitum.

### Diagnostic performance.

To assess the diagnostic performance (Table 2), a total of 1,772 clinical specimens from suspected TB- or NTM-infected patients were collected for a year and applied for the two-step kit. The specimens were mostly pulmonary (*n* = 1,539), including sputum (*n* = 802), bronchial washing (*n* = 569), pleural fluid (*n* = 146), synovial fluid (*n* = 22), and nonpulmonary specimens (*n* = 233). Among the specimens, 8 were positive for AFB by a smear test, and a total of 208 (11.8%) were culture positive either in mycobacteria growth indicator tube (MGIT) broth and/or on Ogawa agar media, including M. tuberculosis (*n* = 62) and NTM (*n* = 146). Since the AFB smear testing presented a very low rate of positive results, the mycobacterial culture result was considered for the comparable reference method regardless of the risk of contamination-mediated false-positive results of the method by growth.

**TABLE 2:** Diagnostic performance of the kit in comparison with MGIT culture results

Specimen type	Total no. of positive specimens	MGIT culture			No. of positive specimens found by BPDx kit:									
		No. of positive specimens grown^*a*^			TB/NTM detection kit			NTM ID kit						
		Total (%)	M. tuberculosis	NTM	Total (% grown)	M. tuberculosis	NTM	Total (% of NTM)	M. intracellulare	M. avium	M. kansasii	M. massiliense	M. abscessus	M. fortuitum
Pulmonary specimens	1,539	194 (12.6)	56	138	91 (46.9)	45	46	36 (78.2)	14	13	5	3	1	0
Sputum	802	143 (17.8)	41	102	59 (41.3)	29	30	22 (73.3)	7	10	2	2	1	0
Bronchial washing	569	49 (8.6)	15	34	31 (63.3)	16	15	14 (93.3)	7	3	3	1	0	0
Pleural fluid	146	2 (1.4)	0	2	1 (50.0)	0	1	0 (0.0)	0	0	0	0	0	0
Synovial fluid	22	0 (0.0)	0	0										
Extrapulmonary specimens^*b*^	233	16 (6.9)	6	10	12 (75.0)	5	7	4 (57.1)	4	0	0	0	0	0
Total	1,772	208 (11.7)	62	146	103 (49.5)	50	53	40 (75.5)	18	13	5	3	1	0

a Bacterial culture was considered positive if the culture was observed either in broth or on solid media.

b Extrapulmonary specimens include cerebrospinal fluid (*n* = 107), urine (*n* = 19), peritoneal fluid (*n* = 2), and others (*n* = 103).

The MTB/NTM detection kit, which is the primary step of the BPDx kit, positively detected 50 M. tuberculosis and 53 NTM cases. Among those 103 positive results, 85 (43 M. tuberculosis) (69.4% of a total of 62 M. tuberculosis culture-positive) and 42 NTM (28.8% of a total of 146 NTM culture-positive) cases corresponded to the results of mycobacterial species culture, and 18 (7 M. tuberculosis and 11 NTM) cases did not. Based on the results, the final sensitivity of the BPDx MTB/NTM detection kit was 40.9% (95% confidence interval [CI], 34.1% to 47.9%), the specificity was 98.9% (95% CI, 98.2% to 99.3%), and the accuracy was 92.0% (95% CI, 90.7% to 93.3%). For M. tuberculosis detection, the sensitivity, specificity, and accuracy were 69.4% (95% CI, 56.4% to 80.4%), 99.6% (95% CI, 99.2% to 99.8%), and 98.5% (95% CI, 97.9% to 99.0%), respectively, and those for NTM detection were 28.8% (95% CI, 21.6% to 36.8%), 99.3% (95% CI, 98.8% to 99.7%), and 93.5% (95% CI, 92.3% to 94.6%), respectively. The diagnostic power was ordinary and similar to that of the widely used PowerCheck MTB/NTM real-time PCR kit (Table 3).

**TABLE 3:** Comparative analytical performance of the BPDx TB/NTM detection kit versus the PowerCheck MTB/NTM real-time PCR kit

Target	Performance	BPDx TB/NTM detection kit		PowerCheck MTB/NTM real-time PCR kit	
		%	95% CI^*a*^	%	95% CI
Total	Sensitivity	40.9	(34.1–47.9)	35.6	(29.1–42.5)
	Specificity	98.9	(98.2–99.3)	99.5	(99.0–99.8)
	Positive predictive value	82.5	(74.4–88.5)	90.2	(81.9–95.0)
	Negative predictive value	92.6	(91.8–93.4)	92.1	(91.3–92.8)
	Accuracy	92.0	(90.7–93.3)	92.0	(90.6–93.2)
M. tuberculosis	Sensitivity	69.4	(56.4–80.4)	64.5	(51.3–76.3)
	Specificity	99.6	(99.2–99.8)	99.8	(99.4–99.9)
	Positive predictive value	86.0	(74.2–92.9)	90.9	(78.7–96.4)
	Negative predictive value	98.9	(98.4–99.2)	98.7	(98.2–99.1)
	Accuracy	98.5	(97.9–99.0)	98.5	(97.9–99.0)
NTM	Sensitivity	28.8	(21.6–36.8)	23.3	(16.7–31.0)
	Specificity	99.3	(98.8–99.7)	99.8	(99.4–99.9)
	Positive predictive value	79.3	(66.8–87.9)	89.5	(75.4–95.9)
	Negative predictive value	93.9	(93.3–94.5)	93.5	(93.0–94.1)
	Accuracy	93.5	(92.3–94.6)	93.5	(92.2–94.6)

a CI, confidence interval.

Notably, five of the undetected NTMs in the primary step were identified as M. avium (*n* = 4) and M. kansasii (*n* = 1) in the secondary step. Among the 53 NTM-positive cases, 40 (75.5%) were identified in the second step for NTM species identification, including 18 M. intracellulare, 13 M. avium, 5 M. kansasii, 3 M. massiliense, and 1 M. abscessus. No cases of M. fortuitum were identified. The remaining 13 cases, which were NTM positive but failed to be identified in the secondary step, were further analyzed by ITS sequencing, and they were mostly Mycobacterium spp., including M. lentiflavum and M. gordonae, rather than the six dominant NTM species of clinical importance.

## DISCUSSION

The identification of causative pathogens for mycobacterial infection is critical not only to improve the outcomes of patients with TB and NTM infections but also to implement proper infection control and prevention strategies. The nucleic acid amplification test is known to be a robust method to apply to clinical specimens, with the major advantages of its sensitivity, short turnaround time, and ease of application to a point-of-care apparatus. In this study, using a nucleic acid amplification test, a new stepwise real-time PCR method for clinical specimens was designed to include (i) detection of M. tuberculosis and NTM species in clinical specimens, and (ii) identification of the top six prevalent NTM species afterward.

The primary step of the method was the distinct design of the M. tuberculosis species, not M. bovis, which is able to lead to false-positive TB for vaccinated patients. For this purpose, a new target specific for M. tuberculosis, the gene for colonic acid biosynthesis glycosyltransferase, was singled out. Analytical performance using the target had diagnostic power as good as the 16S rRNA gene in previous publications (13) and the IS*6110* of the other commercialized kit used for comparison. In addition, the NTM-specific *rpoB* sequences presented unquestionable amplification of mycobacterial species, excluding MTC species, leading to clear-cut conclusions. Molecular diagnostic methods for M. tuberculosis and NTM detection are often designed to amplify both a common target for all mycobacterial species and an MTC-specific target to discriminate M. tuberculosis from all mycobacterial species. Peculiarly, this novel diagnostic method was designed to independently amplify each target for M. tuberculosis and NTM. The diagnostic power to detect either M. tuberculosis or NTM was as good as that of the commercial real-time PCR kit that is widely used in ordinary laboratories in South Korea. The secondary step of the method, which allowed us to identify the top six prevalent NTM species, presented distinctive performance in identifying three-quarters of NTM clinical isolates. The coverage was exactly as we expected in a species prevalence study (12). Rapid identification of the major species of pulmonary pathogens would help proper antimicrobial therapy. Commercialized real-time PCR kits to detect TB and NTM infections often miss a few important NTM species to detect. For instance, the PowerCheck kit does not detect M. fortuitum among all NTM species. Although insufficient incidence of infection by the species in clinical specimens did not allow testing against the clinical samples, the new diagnostic kit presented good detection performance against the reference strain.

Compared to TB, the importance of NTM diagnostics is often neglected, and it has never been a routine laboratory test, although antimicrobial treatment differs by the causative mycobacterial species, and NTM infections are associated with substantial morbidity and mortality across the world (14). Infectious disease caused by NTM has been well characterized in developed countries (15, 16). However, in developing countries, NTM infection is largely undescribed, and patients are improperly treated because the symptoms of NTM infections are often indistinguishable from those of TB. Smear microscopy, pure culture in multiple media, and histopathology methods have critical limitations in differentiating NTM infections and TB. Therefore, the gold-standard culture method to identify NTM species has a high risk of contamination since it takes 2 to 10 weeks, and many NTM species are of environmental origin. Additionally, culture in MGIT and Ogawa agar often results in bacterial growth with doubt about certain influxes of contaminants while preparing for growth. Thus, the specificity of the test is always a matter for NTM detection.

The present two-step method is extremely practical to diagnose TB and NTM infections for dependable data without false positives by vaccination and identification of the NTM species in less than 2 h to help with proper treatment. However, it also has a few limitations. First, human TB cases caused by M. bovis, which are scarce but could occur in some professions in certain countries, could be overlooked. Second, the target NTM species would need to be modified through the regional traits of prevalence. The target design of the kit needs to be modified based on the regional epidemiology study. Third, the set of clinical specimens used to assess the diagnostic power has limited numbers of TB and NTM infection cases. The target hospital, which is a tertiary hospital in the center of the capital city, has a limited prevalence of TB patients. Further evaluation needs to be carried out in a variety of hospitals, including specialized hospitals for TB and NTM patients.

Even though the socioeconomic status of a country is ameliorated, TB remains a public health concern, and the increasing incidence of NTM infection is raised as an additional issue. Thus, proper diagnosis of both infections is important for every country regardless of its socioeconomic status. The developed two-step method with promising results for most of the suspected cases could be a good solution for setting a proper treatment strategy. Additionally, its open design allowing modification of the target NTM species and its application to point-of-care apparatus present potentialities of the kit.

## MATERIALS AND METHODS

### Sample collection and ethics.

A total of 1,772 nonselective and consecutive specimens, including 1,538 pulmonary samples and 233 other samples, were submitted for mycobacterial culture for 1 year between November 2020 and October 2021 in a general hospital located in Seoul, South Korea. The TB-suspected case was defined based on symptoms or signs suggestive of TB following the criteria of the Centers for Disease Control and Prevention (CDC), including a bad cough lasting for more than 3 weeks, which may be accompanied by other respiratory symptoms (shortness of breath, chest pains, and hemoptysis) and/or constitutional symptoms (loss of appetite, weight loss, chills, fever, night sweats, and fatigue). This prospective study was approved by the Institutional Review Board of Gangnam Severance Hospital, Seoul, South Korea (approval number 3-2020-0216).

### Mycobacterium culture and acid-fast bacillus smear test.

To remove undesirable microorganisms in clinical specimens, the same amount of *N*-acetyl-l-cysteine and 1.5% sodium hydroxide (NALC-1.5% NaOH) was added to the liquid clinical specimens. The mixture was homogenized and centrifuged at 3,500 rpm for 15 min, and the sediment at the bottom of the tube was taken to inoculate both solid medium, 3% Ogawa agar (Union lab, Seoul, South Korea), and liquid medium, the BD mycobacteria growth indicator tube (MGIT) (BD BBL, Franklin Lakes, NJ, USA), and to conduct AFB smear microscopy. AFB smear testing was carried out either through the fluorescence staining method using auramine O or auramine-rhodamine or through the Ziehl-Neelsen method using carbol fuchsin and the Kinyoun method. Interpretations were made according to the standards provided by the CDC.

### Mycobacterial genomes used for designing nucleic acid oligonucleotide sequences for PCR.

Reference genomes of target mycobacterial species, i.e., M. tuberculosis H37Rv (GenBank accession no. NC_000962), M. bovis AF2122/97 (GenBank accession no. NC_002945), M. abscessus ATCC 19977 (GenBank accession no. NC_010397), M. intracellulare ATCC 13950 (GenBank accession no. NZ_CP076382), M. kansasii ATCC 12478 (NC_022663), M. massiliense CCUG 48898 (GenBank accession no. NZ_AP014547), M. avium 2285 (GenBank assembly accession no. GCA_000523675), and M. fortuitum ATCC 6841 (GenBank assembly accession no. GCA_001475495), were downloaded from the GenBank database, and the target gene of each species was searched using BLAST against the standard database of nucleotide collection with an option of including or excluding the target organisms.

### DNA preparation.

Total DNA of reference mycobacterial species, including M. tuberculosis H37Rv, M. bovis AF2122/97, M. abscessus ATCC 19977, M. avium 2285, M. intracellulare ATCC 13950, M. kansasii ATCC 12478, M. massiliense CCUG 48893, and M. fortuitum ATCC 6841, was purchased either from the Korea Collection for Type Cultures or from the American Type Culture Collection. From clinical samples, total DNA was extracted by using the PowerPrep TB extraction kit (Kogene Biotech, Seoul, South Korea) according to the manufacturer’s instructions. Briefly, 100 μL of the DNA extraction buffer was added to the sediment pellet, which was obtained from centrifugation after NALC-1.5% NaOH treatment to remove undesirable microorganisms, and the mixture was boiled for 20 min. The supernatant obtained by centrifugation at 13,500 rpm for 5 min was used for PCR.

### Real-time PCR.

The BPDx TB/NTM detection kit and BPDx NTM ID kit were used according to the manufacturer’s instructions along with the Gentier 96E real-time PCR system (Xi’an TianLong Science and Technology, Xi’an, China). It was conducted with the following program: 50°C for 3 min for UDG activation, 95°C for 10 min for initial denaturation, followed by 45 cycles of 95°C for 10 s and 61°C for 30 s for amplification. A PowerCheck MTB/NTM real-time PCR kit (Kogene Biotech, Seoul, South Korea) accompanied by an Applied Biosystems 7500 real-time PCR instrument system (Thermo Fisher Scientific, Waltham, MA, USA) was used as a comparative real-time PCR method according to the manufacturer’s instructions. The following program was used to run the PCR: 95°C for 10 min in 1 cycle, followed by 95°C for 15 s and 60°C for 1-min repeated over 45 cycles. For the samples that showed positive results by using either the BPDx TB/NTM detection kit or the PowerCheck MTB/NTM real-time PCR kit, the detected pathogen was verified through PCR and subsequent Sanger sequencing using a set of primers for internal transcribed spacer (ITS), ITS-F_rrs-1395-1415 of 5′ to 3′, CCCGTCACGTCATGAAAGTC, and ITS-R_rrl-81-62, GAGGCWTATCGCAGCCTCC. The subspecies of M. abscessus were verified by using another set of primers, for rpoB_MABS_F2728-2748 of 5′ to 3′, GACATCATCCTGAACACCCAC, and rpoB_MABS_R3078-3056 of 5′ to 3′, CAGCTTCAGGATGTACATGTAAC (12). Species identification based on the ITS sequence was carried out by BLAST against a homemade database. The database was made to include ca. 280-bp sequence between the 16S and 23S ribosomal RNAs of the >60 Mycobacterium spp., which were extracted from the Entrez Genome database of the National Center for Biotechnology Information (https://www.ncbi.nlm.nih.gov/genome). Quality control of the database was carried out *in silico* by BLAST against a nucleotide collection (nonredundant nucleotide [nr/nt]) of limited taxa.

### Statistical analyses.

For the evaluation of diagnostic power, accuracy, sensitivity, specificity, positive predictive value, and negative predictive value were calculated using Microsoft Excel.
